# Hair cortisol concentrations to picture the dysregulation of the hypothalamic-pituitary-adrenocortical axis in panic disorder

**DOI:** 10.1038/s41598-026-50934-z

**Published:** 2026-05-13

**Authors:** Katja Petrowski, Vanessa Renner, Benedict Herhaus, Rupert Conrad, Clemens Kirschbaum

**Affiliations:** 1 Medical Psychology and Medical Sociology, University Medical Center of the Johannes Gutenberg University of Mainz, Duesbergweg 6, 55128 Mainz, Germany; 2 Department of Psychosomatic Medicine and Psychotherapy, University Hospital Muenster, Muenster, Germany; 3 Department of Psychology, Institute of Biological Psychology, Technical University, Dresden, Germany

**Keywords:** Hair cortisol, Panic disorder, Hypothalamic-pituitary-adrenocortical system, Diseases, Endocrinology, Neuroscience, Physiology, Psychology, Psychology

## Abstract

Panic attacks are characterized by intense feelings of uncontrollable threat and induce experiences of intense stress. As one of the major physiological stress systems of the body, the hypothalamic-pituitary-adrenocortical axis (HPA) may be a key to our understanding of a biological basis of this anxiety disorder. While recent studies have found evidence for short-term HPA changes in panic disorder with flattened cortisol responses to stimulation, no data have been available on tonic, i.e., long-term HPA activity in these patients. The current study therefore investigated the cumulative cortisol incorporation in hair over the period of three months in patients with panic disorder (*n* = 45) and healthy individuals (*n* = 45). Results showed higher hair cortisol concentration in patients with panic disorder compared to healthy controls. The duration and the severity of the disorder was unrelated to hair cortisol concentrations. In subsample analyses, patients with panic disorder and comorbid depression showed no significant differences in hair cortisol concentration compared to patients with pure panic disorder. The present findings do not support to the notion that a hypoactive HPA axis may be an important biological feature of patients suffering from panic disorder. Future studies will have to show in a large sample whether a changed HPA axis reactivity is present and causally related to the development and course of the disorder.

## Introduction

The feeling of fear represents a biologically determined emotional reaction to ensure survival in threatening situations. Anxiety gains pathological value when the feeling does not appear in response to real threats, is experienced as uncontrollable, and leads to avoidance behavior. Patients with panic disorder report sudden, intense and acute anxiety with numerous physical complaints (e.g., racing heart, sweating, tremors) and accompanying cognitions (e.g., fear of losing control, fear of dying, fear of going crazy), see^1^. These panic attack symptoms resemble stress experiences, which is why the HPA axis is of particular importance for panic disorders.

A widely used model that takes into account both the biological and the psychological mechanisms in the development of mental stress disorders is the allostatic load concept by McEwen^2^. It postulates that repeated effects of stressors (“repeated hits”), e.g. in panic attacks and anxiety, lead to repeated activation of the HPA axis. In case of a lack of adaption/habituation to such repeated hits, the stressors may lead to increased cortisol secretion in the short term (prolonged response), which in the long run leads to a decrease of the stress reactivity of the HPA-axis in terms of a blunted cortisol response^3,4^. According to McEwen, the changed HPA axis reactivity would represent a vulnerability factor for the development of anxiety disorders.

Concerning the HPA axis and its basal cortisol concentration in patients with PD, no differences were observed in the cortisol awakening response between patients with panic disorder and healthy individuals^5–10^. Even though higher cortisol levels in patients with panic disorder were reported during certain times of the day or night^11–14^, no differences in cortisol levels have been reported for other periods throughout the afternoon hours of the circadian rhythm^9^.

Many illnesses showed general changes in HPA axis regulations. In patients with panic disorder, hyporeactivity of the HPA axis under psychopharmacological (DEX-CRH;^15^), metabolic stress induction^16^ and psychosocial stress induction (TSST, Trier Social Stress Test,^17^; in PD:^18^ was measured both in saliva^18^ as well as in the blood^19^. When considering the course of the disorder concerning the HPA axis reactivity, no differences between acute and remitted patients with panic disorder were found^9^. However, patients with shorter illnesses showed a significantly stronger HPA axis reactivity than patients with chronic illness (> 2 years;^1,20^).

While spot assessments of cortisol levels in blood or saliva may be informative of the acute HPA status, biomarkers of HPA long-term activity are needed^10,21^. An analysis of the amount of cortisol incorporated into hair provides methodical access to generate such an integral estimate of cortisol production over several months. Hair segment analysis has been used successfully in several clinical samples including depression, post-traumatic stress disorder, or generalized anxiety disorder^21–28^.

In contrast to these clinical conditions, no studies have been available on hair cortisol concentration in patients with panic disorder. Since HPA axis activity has been studied only with short-term cross-sectional measurements in these patients, hair cortisol concentration may inform us whether or not a generally hypoactive HPA axis (as reflected by reduced ACTH and cortisol secretion in response to pharmacological or psychosocial stimulation) is a key feature of panic disorder.

## Results

### Sample characteristics

A description of the *n* = 45 patients with PD with/without agoraphobia and the *n* = 45 healthy controls is given in Table 1. The mean age of the PD group was 35.49 (SD = 15.24), the mean age of the HC group was 31.24 (SD = 12.34) with no significant group differences (*t* (88) = 1.452, *p* = 0.15). Results for clinical measure in the individuals with PD and healthy controls are presented in Table 1.

**Table 1:** Characteristics of the total sample.

	Individuals with panic disorder	Healthy Controls	*t/x*^*2*^	*p*
*Sociodemographics*				
Total, N	45	45		
Females, n (%)	40 (89)	40 (89)	1.000	1.00^a^
Age (years), M (SD)	35.49 (15.24)	31.24 (12.34)	1.452	.15^b^
BMI (kg/m^2^), M (SD)	23.77 (4.32)	22.86 (2.89)	1.161	.25^b^
*Clinical measures*				
PAS total score [0–52]	17.86 (8.73)	5.47 (1.18)	9.438	≤ 0.001^b^
ACQ total score [1–5]	2.16 (.55)	1.38 (.29)	8.341	≤ 0.001^b^
BSQ total score ^1–5^	2.71 (.74)	1.88 (.64)	5.747	≤ 0.001^b^
BDI [0–63]	17.64 (11.59)	4.47 (4.47)	7.116	≤ 0.001^b^

ACQ, Agoraphobic Cognitions Questionnaire; BSQ, Body Sensations Questionnaire; BDI, Beck Depression Inventory; PAS, Panic & Agoraphobia Scale.

^a^Chi-square test.

^b^Independent Student t-test.

### Hair cortisol levels in individuals with PD

**Fig. 1:**
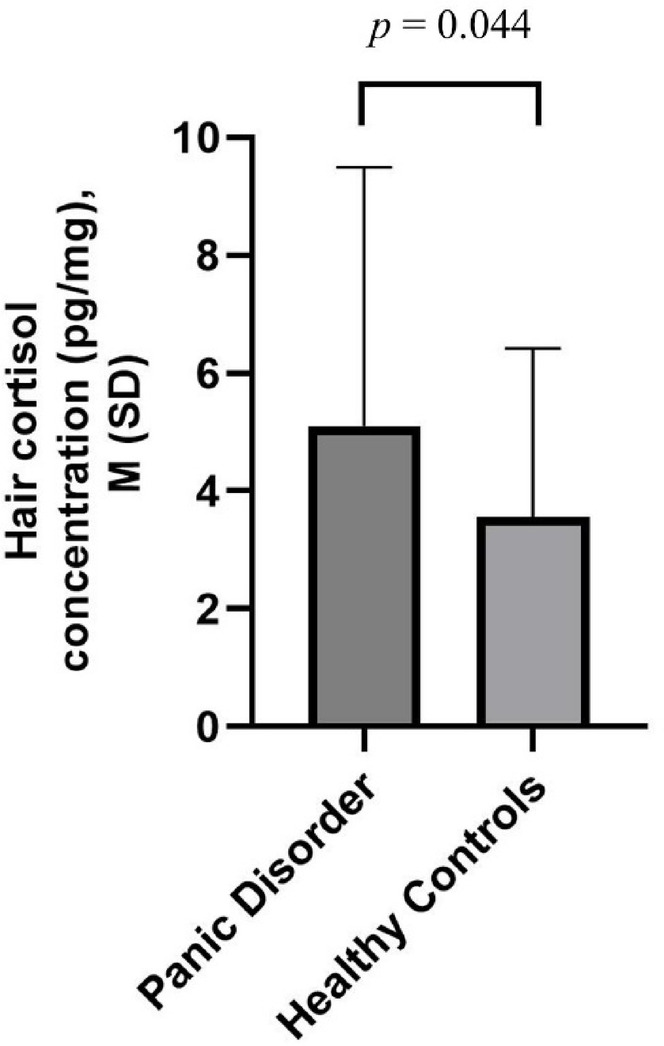
Comparison of hair cortisol concentration (Mean, SD) between individuals with panic disorders and healthy controls.

As shown in Fig. 1, there was significant higher hair cortisol concentration in individuals with PD compared to healthy controls (*t* = 2.039, *p* = 0.044, *d* = 0.43).

### Hair cortisol levels in individuals with PD with and without comorbidity

**Fig. 2:**
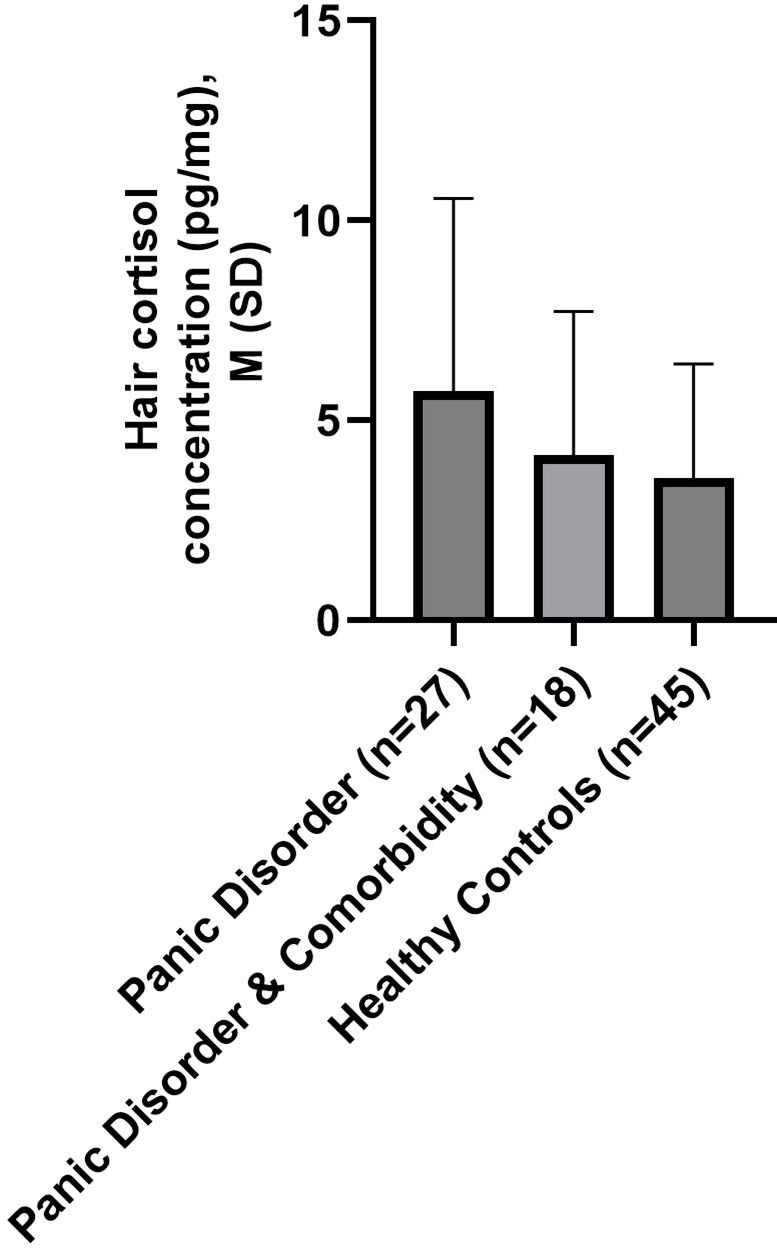
Group Comparisons (Mean, SD) of hair cortisol concentration between individuals with PD, individuals with PD and comorbidity and HC by post-hoc-Test (Bonferroni-Holm corrections).

Hair cortisol concentration between the three groups PD only, PD plus comorbidities, and healthy controls demonstrated significant differences (*F*_(1, 88)_ = 4.158, *p* ≤ 0.05, *η*^*2*^ = 0.045). Post-hoc analyses showed only marginal significant higher hair cortisol concentration in PD only compared to healthy controls (*t* = 2.333, *p* = 0.06, *d* = 0.57; see Fig. 2 and Table 2).

**Table 2:** Results of ANOVA.

	ANOVA			post-hoc-tests					
parameter				(Bonferroni-Holm correction)					
	*F*	*df*	*p*	*PD & Comorbidity vs. PD*		*HC vs. PD*		*HC vs. PD & Comorbidity*	
				*t*	*p*	*t*	*p*	*t*	*p*
Hair cortisol	4.158	2	≤ .05	1.120	.54	2.333	.07	-0.820	.42

HC, healthy controls; PD, Panic Disorder.

### Predictive analysis hair cortisol concentration

The R^2^ for the overall model was 0.204 (adjusted R^2^ = 0.041), indicative for a low goodness-of-fit. PD symptom severity (questionnaire PAS) and duration of disorder were not able to significantly predict hair cortisol concentration in individuals with PD (*F*_(2,40)_ = 0.822, *p* = 0.45).

## Discussion

Patients with panic disorder report anxiety attacks with numerous physical complaints with accompanying catastrophic cognitions^1,2^ which resemble frequent experiences of stress. Therefore, the HPA axis is of particular importance for panic disorders.

However, there is an unclear HPA axis (re)activity in panic disorders over the course of the time or days. Therefore, the hypocortisolism under HPA axis stimulation without baseline differences in the afternoon^8,9^ as well as in the cortisol awakening response^8,18^ is in contrast to the hypercortisolism over the day^12–14^ and at night^11^. These differences might be explicable by cross-sectional snapshots and influencing factors (e.g., panic attacks, daily hassles) using one—time probes during the day and at night^10,21^. Therefore, a different methodological approach was chosen by the cumulative amount of cortisol incorporated in the hair over the period of three months. So far, there are no studies on hair cortisol concentration in patients with panic disorder and the course of the disorder.

For the first time, the present study showed significant higher hair cortisol concentration incorporated over the previous three months in patients with panic disorder compared with healthy controls. These differences in cortisol concentrations might be explicable since the patients with panic disorder experienced panic attacks during the observational period. These results indicate that the patients with panic disorder produce a higher amount of cortisol over the course of three months than healthy controls. However, a reduced responsiveness to acute stimuli, together with elevated cortisol levels during spontaneous panic attacks, may lead to a slightly increased overall cortisol production across the entire month^12^. Past studies found a blunted stress response to the psychosocial stress tests^18^ as well as to pharmacological tests^15,16^ as well as a hypercortisolism over the day^12–14^ and at night^11^.

Based on the allostatic load model^2^ panic attacks can be viewed as “repeated hits” to which the HPA axis does not adapt or habituate. The repeated experience of these stressors may lead to a hypercortisolemic state in the long term (prolonged response;^3,4^). Initial empirical results from a pathophysiological perspective might suggest a change in the sensitivity of adrenocortical ACTH receptors induced by the repeated stimulation^3,9,18^. Based on earlier studies^15,18^, one would propose that patients with panic disorder with comorbidities due to more severe illness show a more reduced reactivity of ACTH following CRH injection^15,20^. Wintermann et al.^20^ also found lower cortisol reactivity in female patients with a chronic course of the disorder (> two years).

However, in the present study the comorbidity, the duration of the disorder and the correspondingly longer experience of panic attacks showed no effect on the concentration of hair cortisol. Interestingly, patients with panic disorder and comorbid depression had significantly lower hair cortisol concentration compared to those with only panic disorder, despite earlier studies suggesting higher cortisol reactivity in patients with comorbid depression due to the depression^15^. These differences between the current study and earlier results might be explicable by the characteristics of the sample. Within the current sample, 34 patients exhibited a chronic disease course (> 1.5 years), while 11 had a more recent onset (≤ 1.5 years). As a result, the small number of patients with recent-onset symptoms and no comorbidities, along with the limited sample size, may explain some of the discrepancies with previous studies. Additionally, while prior studies assessed cortisol reactivity following HPA-axis stimulation, the current results are based on hair cortisol concentration measurements, which reflect cumulative cortisol levels over the past three months. Another limitation is that early adversity was not assessed or controlled for, although it has been shown to influence hair cortisol concentration^29^ and should therefore be addressed in future studies. However, given that patients with panic disorder have been reported to show significantly higher levels of early adversity in self-reports than healthy controls (Bandelow et al., 2004), it appears unlikely that the lower hair cortisol concentration levels in the healthy control group can be explained by early adversity.

Future studies should monitor hair cortisol concentration over an extended period of time in a larger sample and include assessments both before and after psychotherapy^10^. In order to characterize HPA functioning more completely, simultaneous assessments of short-term and long-term HPA biomarkers are needed.

## Methods

### Sample

Hair samples of 50 patients suffering from PD with or without agoraphobia according to the Diagnostic and Statistical Manual of Mental Disorders (DSM-IV;^30^ were collected. The samples were received from patients before participating in a heart rate variability biofeedback training intervention study. Exclusion criteria were specific psychological diseases (e.g., substance abuse, schizophrenia, bipolar or personality disorders), severe chronic physical diseases (e.g., diabetes or cancer), cardiac disorders, psychotropic drugs affecting the central nervous system (e.g., antidepressants) or medication affecting heart rate, pregnancy, and ongoing psychotherapy. Nineteen of the 50 patients were diagnosed with PD with agoraphobia and another 19 had at least one comorbid diagnosis additionally to PD with/without agoraphobia. Seven patients had a comorbid specific phobia (F40.2), eight showed a comorbid depression (F33.0, F33.1, F32.0), and one patient was diagnosed with both specific phobia and depression. One patient was additionally diagnosed with dysthymia (F34.1), one patient with autism (F84.0), and one patient with social phobia (F40.1). Hair samples of healthy subjects were retrieved from a pool of 337 healthy individuals who participated in former studies. The exclusion criterium was, additionally to the ones described for the PD sample, any psychological disease. From this participant pool, healthy subjects were matched with PD patients regarding age and gender.

### Hair cortisol concentration measures and analysis

Hair strands (2–3 mm in diameter) were taken as close as possible to the scalp from the same posterior vertex region. The hair strands were cut into segments of 3 cm length. The determination of hair cortisol concentration was carried out according to the protocol by Steudte et al.^25^ using liquid chromatography coupled with tandem mass spectrometry following the protocol by Gao et al.^31^

### Statistical analysis

All statistical analyses were performed using SPSS version 23 (IBM, Chicago, Illinois). After matching healthy subjects to PD patients regarding gender and age, group differences regarding gender and age were calculated with χ^2^- and t-tests. To assess differences of hair cortisol concentration in PD patients compared with healthy controls, group comparisons were performed using independent t-Test. The hair cortisol concentration were transformed by logarithm naturalis to obtain approximately normal distributions. For further analysis, the group of PD patients was divided into those with PD only and those with additional comorbidities. Univariate analysis of variance (ANOVA) with post hoc tests (Bonferroni-Holm correction) was performed to compare these three groups: 1) healthy controls 2) PD patients and 3) PD patients with comorbidities. A multiple linear regression was calculated to assess if symptom severity (PAS questionnaire) and duration of disorder predict hair cortisol concentration.

## Data Availability

The raw data supporting the conclusions of this article will be made available by the corresponding author upon reasonable request.
